# Prediction of Driving Safety in Individuals with Homonymous Hemianopia and Quadrantanopia from Clinical Neuroimaging

**DOI:** 10.1155/2014/754042

**Published:** 2014-02-05

**Authors:** Michael S. Vaphiades, Lanning B. Kline, Gerald McGwin, Cynthia Owsley, Ritu Shah, Joanne M. Wood

**Affiliations:** ^1^Department of Ophthalmology, School of Medicine, University of Alabama at Birmingham, Birmingham, AL 35294, USA; ^2^Department of Neurology, School of Medicine, University of Alabama at Birmingham, Birmingham, AL 35294, USA; ^3^Department of Surgery, University of Alabama at Birmingham, Birmingham, AL 35294, USA; ^4^Department of Radiology, University of Alabama at Birmingham, Birmingham, AL 35294, USA; ^5^School of Optometry and Vision Science, Queensland University of Technology, Kelvin Grove, QLD 4059, Australia; ^6^Institute of Health and Biomedical Innovation, Queensland University of Technology, Kelvin Grove, QLD 4059, Australia

## Abstract

*Background*. This study aimed to determine whether it is possible to predict driving safety of individuals with homonymous hemianopia or quadrantanopia based upon a clinical review of neuroimages that are routinely available in clinical practice. *Methods*. Two experienced neuroophthalmologists viewed a summary report of the CT/MRI scans of 16 participants with homonymous hemianopic or quadrantanopic field defects which indicated the site and extent of the lesion and they made predictions regarding whether participants would be safe/unsafe to drive. Driving safety was independently defined at the time of the study using state-recorded motor vehicle crashes (all crashes and at-fault) for the previous 5 years and ratings of driving safety determined through a standardized on-road driving assessment by a certified driving rehabilitation specialist. *Results*. The ability to predict driving safety was highly variable regardless of the driving safety measure, ranging from 31% to 63% (kappa levels ranged from −0.29 to 0.04). The level of agreement between the neuroophthalmologists was only fair (kappa = 0.28). *Conclusions*. Clinical evaluation of summary reports of currently available neuroimages by neuroophthalmologists is not predictive of driving safety. Future research should be directed at identifying and/or developing alternative tests or strategies to better enable clinicians to make these predictions.

## 1. Introduction

Homonymous hemianopic and quadrantanopic field loss are widely considered to be incompatible with safe driving in many jurisdictions across the world. However, a number of recent reports have challenged this assumption, suggesting that some individuals with homonymous field defects have the potential for safe driving [1–4], particularly if they adopt compensatory patterns of head and eye movements [3, 4]. Based on this growing body of work, some jurisdictions may reevaluate their policies of prohibiting hemianopic and quadrantanopic persons from driving, and it is likely that input and recommendations from treating neuroophthalmologists or neurologists will have a major role. Their recommendations could be based, at least in part, on the results of an on-road assessment by a certified driving rehabilitation specialist (CDRS) or similarly trained professional [5]. However, clinics and agencies that provide on-road driving evaluations are not widely available in many areas, and, if available, are costly and often not covered by health insurance. There are also concerns about the risk to road safety of taking potentially unsafe drivers on the road, even if the assessment vehicle has dual-brake controls.

An important question is how accurately neuroophthalmologists can predict whether a patient has the potential for safe driving from the clinical information available to them. Given that patients with homonymous field loss generally have neuroimaging studies as part of their clinical evaluation, one possibility might be that such studies of the loci and characteristics of the causative lesion might be of value in determining driving safety. Functional magnetic imaging studies (fMRI) have identified those regions of the brain that are activated by specific driving tasks including busy traffic [6], driver decision-making [7], and undertaking concurrent conversations while driving [8]. In related studies, increased cerebral blood flow in the posterior cingulated gyrus, as measured by 3-dimensional PET, was correlated with the number of crashes in a simulator [9]. A recent study of hemianopic drivers also demonstrated that problems in collision avoidance in a virtual reality simulator were associated with damage in the parietooccipital region and posterior cingulate gyrus in the right hemisphere and the inferior occipital cortex and parts of the occipitotemporal (fusiform) gyrus in the left hemisphere [10]. Other approaches have identified those neuropsychological tests that are most predictive of driving safety and linked these to specific lesion sites. For example, lesions in the prefrontal cortex may impact executive function which has been shown to be highly predictive of older driver safety [11], although there have been no studies that have explicitly linked lesions in a particular brain region with driving performance and safety.

The purpose of this study was to assess whether it is possible to predict which patients have the potential for safe driving from neuroophthalmologists' judgments based upon neuroimaging reports. This study was part of a larger study investigating the driving performance and safety of individuals with homonymous hemianopia and quadrantanopia [2, 3, 12, 13].

## 2. Materials and Methods

### 2.1. Participants

All participants were current drivers or had driven in the last 2 years prior to enrolment in the study, were legally licensed to drive, and had visual acuity of 20/60 or better in at least one eye (vision requirement for licensure in Alabama). Exclusion criteria were Parkinson's disease, multiple sclerosis, Alzheimer's disease, hemiparesis, ocular or neurological conditions resulting in visual field defects (other than hemianopia or quadrantanopia), and lateral spatial neglect as defined by the Stars test [14].

The protocol was approved by the Institutional Review Board for Human Use at the University of Alabama at Birmingham and adhered to the Tenets of the Declaration of Helsinki. After the purpose of the study was explained, participants were asked to sign a document of informed consent before enrolling.

The main study included 22 persons with homonymous hemianopia and eight persons with homonymous quadrantanopic visual field defects (*M* = 52.7 ± 19.8 years). Of the participants, we were able to access the CT/MRI scans of 16 individuals, all of which demonstrated lesions that would cause the homonymous field defects. Twelve of these participants had homonymous hemianopic defects and four had quadrantanopic defects. A detailed description of the visual field characteristics and etiology of brain injury for the participants with hemianopic and quadrantanopic field defects is presented in Table 1. In summary, for the participants with hemianopic field defects, there were four with right hemianopic loss and eight with left hemianopic loss, and five of the twelve had macular sparing. For the participants with quadrantanopia, half had left-sided loss and half right-sided loss, with two with superior loss and two with inferior field loss. The most common underlying aetiology of field loss was stroke (56%), with the remaining causes being trauma, tumor, arteriovenous malformation, and congenital abnormalities.

### 2.2. Driving Assessment

On-road driving performance was assessed under in-traffic conditions in an automatic transmission vehicle (Chevrolet Impala 2007). The driving performance of each participant was assessed under in-traffic conditions along 6.3 miles of noninterstate driving in residential and commercial areas of a city as described previously [2]. Drives were held between 9 a.m. and 3 p.m. to avoid rush hour traffic and were cancelled if it was raining or the road was wet. A CDRS who was also a licensed occupational therapist specializing in vision impairment sat in the front passenger seat of the vehicle; she has 8 years of clinical experience in driving assessment and rehabilitation of patients with a wide variety of medical and neurological conditions. The CDRS evaluated driving performance, had access to a dual brake, and was responsible for monitoring safety and was aware of the medical and functional characteristics of the participants she was evaluating on the road, as is standard practice. Two backseat raters who were masked to the visual field status (i.e., hemianopia/quadrantanopia/normal) and health characteristics of each participant also rated driving safety on a 5-point scale. There was perfect agreement between the CDRS and the backseat evaluators in terms of determining which of the drivers passed or failed the driving assessment [12], which provides important validation regarding the reliability of the CDRS's judgments with respect to safe driving (the study's main driving performance dependent variable).

Each drive began by participants completing a series of basic driving maneuvers in a parking lot to ensure that they had adequate vehicle control and to become familiar with the vehicle. Once the participant exhibited adequate vehicle control, the on-road driving evaluation began, starting in quiet city streets in a residential neighbourhood and then proceeding to busier roads. The CDRS used a 5-point rating system to assess different components of driving performance, as well as to derive an overall rating of performance, where (1) *driving was so unsafe that the drive was terminated*; (2) *driver exhibited a couple of unsafe maneuvers but did not reach the level of drive termination*; (3) *driving was unsatisfactory but not unsafe at that time given the traffic circumstances*; (4) *driver exhibited a few minor driving errors*; and (5) *there were no obvious driving errors* [12]. Scores of 1 and 2 were classified as failing the driving assessment and being unsafe to drive, while scores of 3, 4, and 5 were considered to be passes.

### 2.3. Motor Vehicle Collision Data

Information regarding police reported motor vehicle collisions was obtained from the Alabama Department of Public Safety for the period from January 2002 to December 2007. For those patients diagnosed after January 2002, only those collisions which occurred after diagnosis were considered. Each collision was classified as to whether, according to the police officer at the scene who completed the report, the study participant contributed to the collision and was deemed at-fault.

### 2.4. Clinical Evaluation of the Images

Sixteen participants with homonymous hemianopic/quadrantanopic field loss had CT/MRI scans available, the results of which were summarized in a report by a neuroradiologist which provided information regarding the site and extent of the lesion. The neuroradiologist was masked to all other clinical characteristics of the patients. Two senior experienced neuroophthalmologists independently reviewed the neuroimaging report for each participant. The neuroophthalmologists were masked to all other clinical characteristics of participants. Based upon their clinical experience regarding the functional impact of the size and the site of a given lesion along the visual pathways, each neuroophthalmologist judged whether the participant would be either potentially safe or unsafe to drive. While both size and location were important, the lesions considered to be more problematic were larger lesions that extended into the visual association areas.

## 3. Results

Table 2 shows the number of participants predicted to be either safe or unsafe to drive by the two neuroophthalmologists. The level of agreement between the neuroophthalmologists in their driving safety predictions was only fair (*κ* = 0.28), with neuroophthalmologist 1 predicting that 9/16 (56%) of the patients would be unsafe, while neuroophthalmologist 2 predicted that only 5/16 (31%) of the patients would be unsafe to drive.

Table 3 shows the ability of the respective neuroophthalmologists to correctly predict safe and unsafe outcomes, defined in terms of the potential for either safe or unsafe driving performance as assessed under in-traffic conditions, all crashes, as well as at-fault crashes. Importantly, while there was variation in the predictive ability between the two neuroophthalmologists, it is clear that neither was able to accurately predict either safe or unsafe driving as defined by the various driving outcome measures. The correct proportion of agreement, calculated here as total proportion of correct safe and unsafe predictions, ranged from as low as 31.3% for neuroophthalmologist 1 against on-road driving outcomes to 62.5% for neuroophthalmologist 2 against at-fault crashes, with corresponding *κ* coefficients ranging from as low as −0.29 (less than chance) to 0.04 (poor).

## 4. Discussion

In this study we investigated how well two experienced neuroophthalmologists could predict driver safety from the neuroimaging reports based on CT/MRI scans of patients with a range of hemianopic or quadrantanopic field defects. This is an important question because on-road driving assessments are not widely available and have cost and safety implications and may not be necessary if clinical assessment of causative lesions could accurately predict potential driving safety. The results of this study suggest that two experienced neuroophthalmologists were not able to accurately predict the potential for safe driving—determined through both recording of prospective state-recorded at-fault MVC, and a standardized on-road driving assessment.

Importantly, there was also a lack of agreement between the two neuroophthalmologists in terms of their clinical predictions of participants' driving safety, with neuroophthalmologist 1 predicting much higher levels of unsafe driving than did neuroophthalmologist 2. These results may reflect in part differences in experience levels of the neuroophthalmologists in treating a range of patients with homonymous hemianopia but more importantly reflect the lack of clear evidence on which to make these judgments about driving safety based on currently available clinical tools.

**Figure 1:**
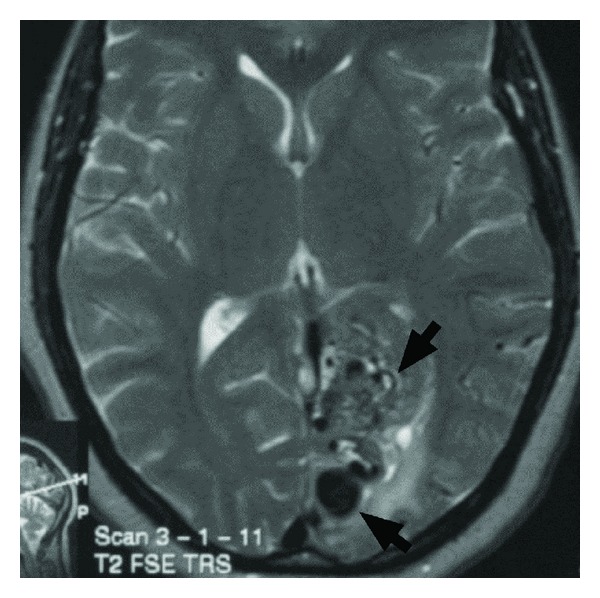
Axial T2 MRI demonstrates a large left parietooccipital arteriovenous malformation yet the patient was found to be a safe driver.

**Figure 2:**
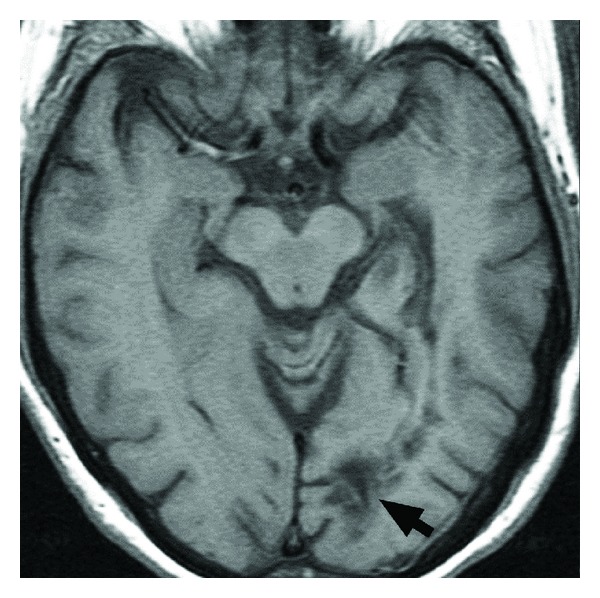
Axial T1 MRI shows evidence of a small left parietooccipital infarction yet the patient was determined to be an unsafe driver.

Our results demonstrate that driving safety cannot be accurately predicted by a subjective, clinical evaluation of currently available CT/MRI scans. The challenge faced by clinicians in making these judgements is illustrated by the scans of two of the patients included in this study.  Figure 1 shows a large left parietooccipital arteriovenous malformation in a patient who was shown to be a safe driver while Figure 2 shows a patient with a small left parietooccipital infarction who was subsequently shown to be unsafe to drive.

The finding that medical specialists are unable to predict the driving safety of their patients from available clinical imaging data is in accordance with previous studies. For example, it has been reported that the potential for safe driving in individuals with Parkinson's disease could not be accurately predicted from either disease rating scales [15] or the opinion of the treating neurologist [16]. Our finding that knowledge of the size and location of the causative lesion does not allow accurate prediction of driving safety, even by experienced clinicians, should not perhaps be surprising given that driving is likely to involve a number of regions of the brain and the complex connections between them. While imaging studies do suggest that certain regions of the brain are linked with specific aspects of driving performance, the results are not clear-cut and there are differences in results between studies for a number of methodological reasons. In addition, while a particular brain region may be linked to some aspect of driving performance, this may not necessarily result in a driver being unsafe.

The results of this study should be considered in light of study limitations. The number of participants was relatively small which limits the generalizability of the findings. In addition, we did not include any patients who failed to recover from their brain injuries sufficiently to be able to return to driving and other visual activities of daily living, all lived independently in the community, and were current drivers, or had driven within the previous two years. The neuroophthalmogists in this study also did not view the neuroimages directly, but rather, as is the case in standard clinical practice, based their decisions on driving safety on summaries of the images made by a neuroradiologist. Nevertheless, the study has a number of unique features in that it is the first to investigate the relationship between clinical evaluation of summary reports of imaging data provided by a neuroradiologist and actual on-road driver performance and crash risk in patients with homonymous hemianopic or quadrantanopic field defects; these included well-validated driving measures including prospective state-recorded MVC data and on-road driving performance as assessed using a standardized route and rating scales. In addition, both of the neuroophthalmologists were masked to the clinical characteristics of participants (except for the imaging results) as well as their MVC data and the outcomes of the driving assessment and undertook their assessments independently of one another to avoid the potential for bias. While information about cognitive status may be available to clinicians, we chose not to include this when determining the clinical judgments of driving safety as we wished to include only those objective clinical measures that are typically available to all clinicians.

## 5. Conclusions

The findings of this study suggest that clinical information on neuroimaging, as is available in standard clinical practice in managing patients with hemianopia and quadrantanopia, is not sufficient for neuroophthalmologists to predict driving safety. It is possible that driving ability may have been more accurately predicted by review of all available data including symptoms, clinical signs, and visual field data, as well as the images; however, as discussed above, previous studies for other populations suggest that this is unlikely [16]. The goal of future research should be to identify and/or develop alternative tests to enable clinicians to better make these predictions.

## Figures and Tables

**Table 1:** Visual field characteristics and etiology of brain injury of the participants.

Participant #	Age (years)	Visual field loss	Etiology^1 ^	Verbatim report by neuroradiologist regarding the CT scan/MRI (size and location of the brain injury)	Years since injury
110	57	Left incomplete homonymous hemianopia with no macular sparing	CVA^2^	Right PCA fusiform aneurysm abuts right optic tract. Right corona radiata infarct that is probably involving the right optic radiation or the LGN	2

115	79	Right incomplete homonymous hemianopia with macular sparing	CVA—left mesial occipital lobe	Old left occipital infarct involving the pole and most of the calcarine cortex	1

118	34	Right incomplete homonymous hemianopia with macular sparing	Arteriovenous malformation—left occipital lobe	Left occipital and medial temporal arteriovenous malformation with oedema surrounding it	17

135	41	Left complete homonymous hemianopia with no macular sparing	Trauma—multiple incidents of trauma associated with boxing career and assault	Probable right occipital encephalomalacia	>10

137	77	Left complete homonymous hemianopia with no macular sparing	CVA	Right posterior cerebral artery infarct involving most of the occipital lobe and part of medial temporal lobe	6

142	83	Left incomplete homonymous hemianopia with macular sparing	CVA	Old right posterior cerebral artery infarct sparing occipital pole; developed huge right posterior cerebral artery infarct	4

146	25	Left incomplete homonymous hemianopia with macular sparing	Tumor—craniopharyngioma treated by resection and radiation	Suprasellar craniopharyngioma compressing optic chiasm; oedema extends into optic tract bilaterally but right more than left involvement	1

150	31	Left complete homonymous hemianopia with no macular sparing	Right temporal lobectomy as treatment for epilepsy following trauma	Right temporal encephalomalacia due to craniotomy that extends into right optic tract and in optic radiation	10

151	66	Left incomplete homonymous hemianopia with macular sparing	CVA associated with cardiac surgery	Right occipital haematoma from AVM; oedema extended into parietal and temporal lobe radiations at the time but later resolved	2

154	43	Right incomplete homonymous hemianopia with no macular sparing	Trauma—parietal and occipital fractures; subarachnoid haemorrhage, from motor vehicle collision	Left posterior-inferior temporal encephalomalacia likely involving the left optic radiation; no occipital cortex involvement	7

158	55	Left complete homonymous hemianopia with no macular sparing	CVA associated with cardiac surgery	Right posterior cerebral infarct that involves medial temporal lobe and anterior aspect of the occipital lobe but partially spares the occipital lobe	3

159	77	Right incomplete homonymous hemianopia with no macular sparing	CVA—left occipital lobe	Left occipital infarct sparing much of the calcarine cortex	5

102	42	Right complete superior quadrantanopia	CVA secondary to vasospasm	Old left occipital and medial temporal lobe infarct (posterior cerebral artery) occipital lobe spared; right anterior temporal and superior frontal encephalomaacia; right postcentral gyrus old infarct	2

106	69	Left incomplete superior quadrantanopia	CVA—right medial temporal lobe and right external capsule	Right internal capsule posterior limb and uncus acute infarct that probably involves right optic tract	2

149	28	Right incomplete inferior quadrantanopia	Left parietal lobe brain tumor	Left parietal encephalomalacia due to tumour resection which extends into parietal optic radiation but spares occipital pole; no temporal lobe involvement	13

152	57	Left incomplete inferior quadrantanopia	Congenital brain abnormality	Small old right occipital infarct spares the occipital pole	Congenital

^1^If brain loci information is not listed in Table 1, it was not available in the medical record.

^2^Cerebral vascular accident.

**Table 2:** Predictions of driver safety by the two neuroophthalmologists.

	Neuroophthalmologist 2	
	Safe	Unsafe
Neuroophthalmologist 1		
Safe	6	1
Unsafe	5	4

**Table 3:** The ability of the two neuroophthalmologists to predict a range of driving outcomes.

Driving outcome	Safety rating	Neuroophthalmologist 1			Neuroophthalmologist 2		
		Safe	Unsafe		Safe	Unsafe	
On-road driving assessment	Safe	4	8		8	4	
	Unsafe	3	1		3	1	
	Proportion agreement			31.25%			56.25%

All crashes	Safe	4	6		7	3	
	Unsafe	3	3		4	2	
	Proportion agreement			43.75%			56.25%

At-fault crashes	Safe	6	7		9	4	
	Unsafe	1	2		2	1	
	Proportion agreement			50%			62.5%
